# One-year survival and resource use after critical illness: impact of organ failure and residual organ dysfunction in a cohort study in Brazil

**DOI:** 10.1186/s13054-015-0986-6

**Published:** 2015-06-25

**Authors:** Otavio T. Ranzani, Fernando G. Zampieri, Bruno A. M. P. Besen, Luciano C. P. Azevedo, Marcelo Park

**Affiliations:** Intensive Care Unit, Emergency Medicine Discipline, Hospital das Clínicas, University of São Paulo, Rua Dr. Enéas de Carvalho Aguiar, 255, 5th floor, Room 5023, São Paulo, 05403-010 Brazil; Research and Education Institute, Hospital Sirio-Libanes, São Paulo, Brazil

## Abstract

**Introduction:**

In this study, we evaluated the impacts of organ failure and residual dysfunction on 1-year survival and health care resource use using Intensive Care Unit (ICU) discharge as the starting point.

**Methods:**

We conducted a historical cohort study, including all adult patients discharged alive after at least 72 h of ICU stay in a tertiary teaching hospital in Brazil. The starting point of follow-up was ICU discharge. Organ failure was defined as a value of 3 or 4 in its corresponding component of the Sequential Organ Failure Assessment score, and residual organ dysfunction was defined as a score of 1 or 2. We fit a multivariate flexible Cox model to predict 1-year survival.

**Results:**

We analyzed 690 patients. Mortality at 1 year after discharge was 27 %. Using multivariate modeling, age, chronic obstructive pulmonary disease, cancer, organ dysfunctions and albumin at ICU discharge were the main determinants of 1-year survival. Age and organ failure were non-linearly associated with survival, and the impact of organ failure diminished over time. We conducted a subset analysis with 561 patients (81 %) discharged without organ failure within the previous 24 h of discharge, and the number of residual organs in dysfunction remained strongly associated with reduced 1-year survival. The use of health care resources among hospital survivors was substantial within 1 year: 40 % of the patients were rehospitalized, 52 % visited the emergency department, 90 % were seen at the outpatient clinic, 14 % attended rehabilitation outpatient services, 11 % were followed by the psychological or psychiatric service and 7 % used the day hospital facility. Use of health care resources up to 30 days after hospital discharge was associated with the number of organs in dysfunction at ICU discharge.

**Conclusions:**

Organ failure was an important determinant of 1-year outcome of critically ill survivors. Nevertheless, the impact of organ failure tended to diminish over time. Resource use after critical illness was elevated among ICU survivors, and a targeted action is needed to deliver appropriate care and to reduce the late critical illness burden.

**Electronic supplementary material:**

The online version of this article (doi:10.1186/s13054-015-0986-6) contains supplementary material, which is available to authorized users.

## Introduction

The effects of critical illness on patients’ lives do not cease after intensive care unit (ICU) discharge [1–9]. Patients who survive ICU stays have higher hospital and post-hospital mortality than other patients and the general population [2, 10]. Furthermore, ICU survivors are at risk of physical, cognitive and mental health consequences [11–13]. Post-ICU mortality seems to be greatest during the first month after ICU discharge, suggesting that even hospital discharge is inaccurate for measuring the impact of critical illness on outcomes [1, 2, 14, 15]. Furthermore, increased health resource use by survivors of critical illness have been described recently in developed countries, representing a substantial component of resource use in the health care system [16–22].

The burden of organ failure, exacerbated inflammatory response and other events (e.g., endothelial activation, physiological derangements) that occur during the acute phase of critical illness are clearly associated with poor short-term outcomes, as captured by ICU and hospital mortality. In contrast, the extent to which these residual effects might influence long-term outcomes is uncertain [1, 14, 23–27]. The cumulative [25] and peak maximum organ failure [26] were associated with long-term mortality; however, in a large cohort of patients with septic shock, the impacts of these factors seemed to be strongest only early after admission [28]. The starting point for describing long-term outcomes is fundamental [1, 29, 30], and the majority of studies have usually started their follow-up periods at ICU admission, including ICU death as a long-term prognosis and perhaps attenuating the impact of organ failure on post-ICU outcomes [1, 26, 29, 30]. In a further step, researchers in a recent study differentiated the determinants of short- and long-term survival after critical illness [1]. Considering the dynamic process of critical illness, this approach is fundamental for the prognostication and discussion of treatment plans, for research and for prevention from both the patient and health system perspectives [1, 26, 31].

The time point of ICU discharge is an intermediate phase that represents an enormous gap in care, and it could be essential to long-term outcomes [8, 29]. Indeed, it is a rich moment for actions (e.g., early rehabilitation) to reduce early complications and to plan treatment goals before hospital discharge [7]. We therefore aimed to evaluate the impacts of organ failure and residual dysfunction on 1-year survival and health care resource use with ICU discharge as the starting point. We hypothesized that the impact of organ dysfunction on prognosis would be more pronounced during the first 90 days, with decreasing impact during the 1 year follow-up period. As secondary endpoints, we evaluated the impact of organ dysfunction on health care use at a public tertiary service in Brazil.

## Methods

### Population

We conducted a historical cohort study that included all patients admitted to a general adult ICU at a tertiary teaching hospital in São Paulo, Brazil, between January 2003 and December 2008. We analyzed patients who were discharged alive from the ICU following at least a 72-h stay. This criterion was chosen because these patients are prone to higher post-ICU mortality than other patients [32]. For patients with multiple ICU admissions, only the first admission was recorded.

### Health care system

Our ICU is located in the Hospital das Clínicas, the largest health care complex in Latin America. At the time the study was conducted, it consisted of 7 specialized institutions with a total of 2,400 beds. There were also one rehabilitation hospital and one long-term acute care facility.

### Our intensive care unit

Our unit is a mixed ICU with seven beds. This unit follows current standard of care practices, including sedation, nutritional, mechanical ventilation and hemodynamic monitoring protocols. An intensivist was available on site 24 h per day, 7 days per week (24/7). The staff consisted of one senior physician, one critical care fellow and three residents from the internal medicine program. At night, there was one senior physician and one resident. The health staff consisted of two nurses and three nursing assistants on a 24/7 schedule, in addition to a respiratory therapist who was on a 12/7 schedule [33]. The unit was a closed unit and received patients from the entire health care complex, although the majority of patients came from the Emergency Department and the medical wards. Patients who underwent cardiothoracic surgery, transplants and elective surgery (e.g., neurosurgical, vascular), as well as trauma and burn patients, are usually admitted to other specialized ICUs in the complex.

This study protocol followed the statements of the Declaration of Helsinki. The hospital system’s institutional review board [Comissão para Análise de Projetos de Pesquisa (CAPPesq)] reviewed and approved this study (CAPPesq protocol number 107443). The requirement for written informed consent was waived because there was no intervention; we used only a database that guaranteed patient confidentiality.

### Data collection

All of the data were recorded prospectively with a computerized physician order entry system. The admission data included patient age, reason for admission, physiological data and Acute Physiology and Chronic Health Evaluation II (APACHE II) score [34]. Organ dysfunction was measured daily using the Sequential Organ Failure Assessment (SOFA) score [35], which includes six types of organ dysfunction in a numeric chart. The daily SOFA score, discriminated by each system evaluated, was recorded. The neurological SOFA score was calculated based on the last evaluation available done when the patient was not sedated. For unique missing values (e.g., if serum bilirubin value was not available), we used the mean value calculated based on the preceding results and the results after the missing value [36]. Our unit uses the SOFA score daily in clinical rounds, and we provide monthly training sessions in its use. We observed only 2 % unique missing values for SOFA scores in our study.

### Organ dysfunction definitions

Total SOFA score was calculated as the sum of each organ score daily during the ICU stay of each patient. The maximum SOFA score was the highest score for the total SOFA during the ICU stay. Organ failure was defined as a value of 3 or 4 for its corresponding component of the SOFA score [8]. Residual organ dysfunction was defined as a score of 1 or 2 [8, 37].

### Follow-up

The patients were followed for 1 year after their ICU discharges using the electronic system available at the Hospital das Clínicas. The electronic system is used for administrative purposes in all services at the health care complex, and every procedure, visit, laboratory examination, vital sign and other data gathered during hospitalization or outpatient visits is compulsorily recorded along with the date and a unique identifier. The health care complex has a policy of following its patients, and, as a general rule, at least one scheduled visit is required for the patient to be discharged. A proportion of these patients attended an intensive care follow-up clinic managed by our ICU that was active from 2008 to 2011.

### Outcomes

The primary endpoint for this study was 1 year survival after ICU discharge. We also evaluated the burden of critical illness 1 year after hospital discharge at the health care complex, including visits to the Emergency Department; outpatient clinic visits; hospital readmissions; and other resource care use issues, such as consultation with psychiatric and psychological services, rehabilitation consultations (physiotherapy, nutrition and speech therapist visits), day hospital use and the number of complementary examinations performed.

### Statistical analysis

Continuous data are presented as mean ± standard deviation or as median and interquartile range, as appropriate. Categorical variables are shown as percentages. For categorical variables, Fisher’s exact test or a χ^2^ test was used; for continuous variables, an unpaired *t* test or the Mann–Whitney *U* test was used if the data were normally or non-normally distributed, respectively.

To evaluate the pattern of SOFA score evolution over the ICU stay, we fit a generalized linear mixed model including the SOFA score at admission (D1) and sequentially on the third ICU day (D3) and at 72 (D − 3), 48 (D − 2) and 24 (D − 1) hours before ICU discharge, accounting for repeated measures correlation. To overcome overlapping of measurements for patients who stayed less than 6 days in the ICU, we maximized the time points for each patient, choosing the D1, D − 2 and D − 1 points for patients who stayed in the ICU for 3 days and D1, D − 3, D − 2 and D − 1 for patients who stayed in the ICU for 4 or 5 days. We conducted a sensitivity analysis defined post hoc, including only patients who stayed in the ICU for at least 6 days.

**Fig. 1:**
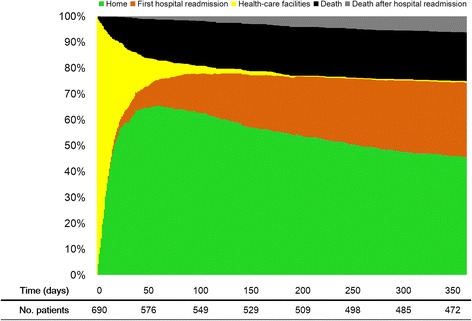
Health care use and survival for 690 patients at 1 year after Intensive Care Unit discharge. Hospital readmission was considered to be major resource use, and, after the first rehospitalization, the patient was truncated in this category; otherwise, the patient was put into the death category if the patient died

The Kaplan-Meier method was used to plot crude survival curves according to the number of organ dysfunctions and was compared using the log-rank test. We present survival and health care use with the same figures, as previously described by Prescott et al. [20]. In the Fig. 1, we plot a survival curve for mortality and a cumulative curve for hospital readmission.

The modeling of long-term outcomes is affected by the statistical model applied. Considering that critical illness has a dynamic effect on outcomes, following the inflammatory response, physiological disturbances and the complex scenarios involved in critical illness, the Cox proportional hazards (PH) model, which is the most frequently used model, might not be accurate, because it relies on the assumption that the prognostic factors have constant hazard ratios over time and that continuous predictors present a linear function with the estimated risk. To address these issues, we fit flexible extensions of a Cox model that have been used with advantageous results [14, 28, 38, 39]. Therefore, we used a flexible Cox model allowing for non-linear relationships between continuous predictors and survival and addressing time-dependent covariates. The outcome was 1-year survival, and the main exposure was the SOFA score. To address potentially confounding factors, we ran a multivariate model, and variables were selected after bivariate analysis using a Cox PH model for the variables described in Table 1. We selected variables associated with 1 year survival (*P* < 0.250). To choose the final multivariable model, we used the Akaike Information Criterion (AIC), looking for parsimonious models with the lowest AIC values [28] through a backward procedure. For each variable, we tested the PH assumption (through inspection of Schoenfeld residuals) and linearity between covariates and the log of hazard ratios. Variables that fulfilled both assumptions were entered as they would be into a traditional Cox PH model. We did not prespecify effect modifiers in our analysis. For continuous variables that did not fulfill the required assumptions, we applied a penalized smoothing spline function. The optimal degree of freedom for the spline was chosen by minimizing the AIC value [40]. Continuous variables were centered on the mean value. ICU length of stay was log-transformed because of its positively skewed distribution. To evaluate the discrimination of the final model, we used time-dependent receiver operating characteristic (ROC) curves. Collinearity was assessed through the variance inflation factor (VIF), and a VIF value greater than 2.5 was considered to be suggestive of collinearity. To address losses to follow-up in the Cox regression, we censored patients lost to follow-up, assuming non-informative censoring. We observed missing data for only 2 % of SOFA scores, for which we calculated mean values between previous and consecutive days around the missing values [36]. To estimate the hazard ratio over time, we used the approach suggested by Harrell [41], repeatedly reestimating the Cox regression model within time intervals. The log hazard ratios were plotted against the mean failure or censoring time within the interval [41]. We conducted a prespecified sensitivity analysis for patients without organ failure at ICU discharge. We employed the same analytical strategy reported above to evaluate the impact of residual organ dysfunction on 1-year survival.

**Table 1:** Characteristics of the patients included in the study

Variables	All patients (*n* = 690)
Age, yr, mean (SD)	50.3 ± 19
Male sex, *n* (%)	358 (52 %)
APACHE II score	16 [10–21]
Type of admission, *n* (%)	
Medical	550 (80 %)
Surgical	140 (20 %)
Comorbidities	
Number of comorbidities, *n* [IQR]	1 [0–2]
Hypertension, *n* (%)	346 (50 %)
Diabetes, *n* (%)	149 (22 %)
COPD, *n* (%)	59 (9 %)
Heart failure, *n* (%)	110 (16 %)
Chronic kidney disease, *n* (%)	113 (16 %)
AIDS, *n* (%)	18 (3 %)
Cancer, *n* (%)	71 (10 %)
Reason for admission, *n* (%)	
Acute respiratory failure	220 (32 %)
Shock	125 (18 %)
Septic shock	111 (16 %)
CNS disorder	78 (11 %)
Monitoring	73 (10 %)
Post-operative period	48 (7 %)
Gastrointestinal diseases	50 (7 %)
Electrolyte disturbances	25 (4 %)
Acute kidney injury	23 (3 %)
Trauma	22 (3 %)
Support during ICU stay, *n* (%)	
Mechanical ventilation	510 (74 %)
Renal replacement therapy	102 (15 %)
Vasopressors	281 (41 %)
SOFA score, median [IQR]	
At admission	4 [2–7]
Maximum	6 [3–9]
At ICU discharge	2 [1–3]
At least one organ failure,^a^ *n* (%)	
At admission	360 (52 %)
Maximum	428 (62 %)
At ICU discharge	129 (19 %)
ICU length of stay, days	
Mean ± SD	10 ± 9
Median [IQR]	7 [4–11]
Albumin at discharge (g/L)	27 [23–31]

*APACHE II* Acute Physiology and Chronic Health Evaluation II, *CNS* Central nervous system, *COPD* chronic obstructive pulmonary disease, *ICU* Intensive Care Unit, *IQR* interquartile range, *SD* standard deviation, *SOFA* Sequential Organ Failure Assessment

^a^Organ failure was defined as a value of 3 or 4 in a corresponding component of the SOFA score

Health care burden was assessed until the first year after ICU discharge. For the analysis, we conducted a prespecified sensitivity analysis by separating the associated burden, which occurred within the first 30 days after hospital discharge [42, 43].

All of the analyses were performed with R software, version 3.0.2. A *P* value of 0.05 was considered to be significant for all comparisons.

## Results

### Baseline characteristics

Of the 1462 patients admitted to the ICU during the study period, 690 patients were discharged alive after a stay of at least 72 h and were therefore included in the present analysis (Additional file 1: Figure S1). Demographic data, reasons for admission, illness severity and other characteristics of the study population are described in Table 1.

### Organ failure and 1-year survival

Four hundred twenty-eight patients (62 %) experienced at least one organ failure during their ICU stay. The time to present the greatest number of organ failures was less than 24 h for 284 patients (65 %). Ninety percent of our study sample demonstrated their greatest burden of organ dysfunction within the first 5 days of ICU admission.

Fifty patients (7 %) were lost to follow-up at 1 year. The general characteristics of the patients who were lost to follow-up were comparable to those of the general population (Additional file 1: Table S1). ICU mortality was 26 %. Following ICU discharge, hospital mortality for the index hospitalization was 18 % (Table 3). For those patients discharged from the hospital, 1-year mortality was 9 %, totaling 27 % of 1-year mortality for patients discharged alive after a 72-h ICU stay.

The survival curve is shown in Fig. 1. Compared with other patients, patients alive after 1 year had lower SOFA values at ICU admission, on the third day after admission, 72 h before discharge, 48 h before discharge and at discharge (Additional file 1: Figure S2A). Similar results were found for patients who stayed at least 6 days in the ICU (Additional file 1: Figure S2B). The maximum SOFA score {6.0 [95 % confidence interval (CI), 5.7–6.4] vs. 8.3 [95 % CI, 7.6–8.9]; mean difference, −2.2 [95 % CI, −2.7 to −1.5]; *P* < 0.001} and mean SOFA score (3.2 [95 % CI, 3.0–3.4] vs. 4.7 [95 % CI, 4.3–5.1]; mean difference, −1.5 [95 % CI, −1.9 to −1.1]; *P* < 0.001) also differed between survivors and non-survivors.

**Fig. 2:**
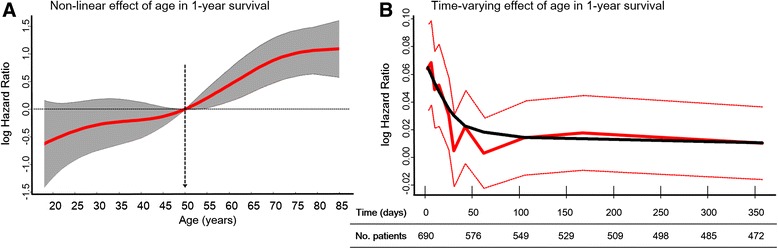
Non-linear effect (**a**) and time-varying effect of age (**b**) on the log of the hazard ratio (HR) for 1-year survival following Intensive Care Unit discharge from the multivariate flexible Cox model. Panel (**a**) shows the log HR for age values, modeled through spline terms with 50 years old as the reference category (mean value). Panel (**b**) shows the time-varying effect of age reestimated within time intervals over 1 year. In (**a**), the *solid red line* denotes the estimated log HR and the *gray region* denotes the 95 % confidence interval. The *black dashed line* with *arrow* denotes the reference value. In (**b**), the *bold solid red line* represents the estimated log HR over time, and the *thin red lines* denote the 95 % confidence intervals. The *solid black line* represents the super-smoothed version of the log HR over time. Age is measured in years. The *y*-axis is in natural log scale; therefore, we present some examples of HRs to clinical interpretation: log (−1) = HR of 0.37, log (−0.5) = HR of 0.60, log (0) = HR of 1, log (1) = HR of 2.7 and log (2) = 7.4

**Fig. 3:**
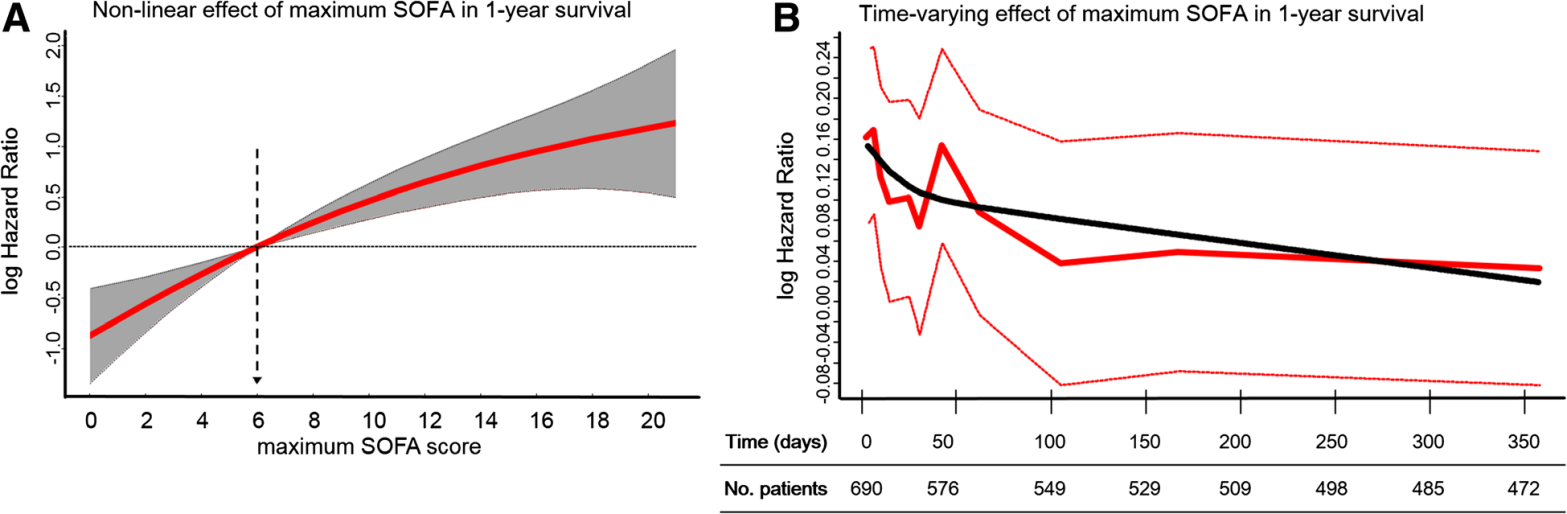
Non-linear effect (**a**) and time-varying effect of maximum Sequential Organ Failure Assessment (SOFA) score (**b**) on the log of the hazard ratio (HR) for 1-year survival following Intensive Care Unit discharge from the multivariate flexible Cox model. Panel (**a**) shows the log HR for maximum SOFA values modeled through spline terms, with the SOFA score of 6 points as the reference category (mean value). Panel (**b**) shows the time-varying effect of maximum SOFA score, reestimated within time intervals over 1 year. In panel (**a**), the *solid red line* denotes the estimated log HR and the *gray region* denotes the 95 % confidence interval. The *dashed black line* with *arrow* denotes the reference value. In panel (**b**), the *bold solid red line* represents the estimated log HR over time and the *thin red lines* denote the 95 % confidence intervals. The *solid black line* represents the super-smoothed version of the log HR over time. The *y*-axis is in natural log scale; therefore, we present some examples of HRs to clinical interpretation: log (−1) = HR of 0.37, log (−0.5) = HR of 0.60, log (0) = HR of 1, log (1) = HR of 2.7 and log (2) = 7.4

Increasing numbers of organ failures had an additive negative impact on 1-year survival (*P* < 0.001 by log-rank test) (Additional file 1: Figure S3). Variables selected to be included in the Cox model were age, sex, arterial hypertension, chronic obstructive pulmonary disease, heart failure, chronic kidney disease, cancer, APACHE II score, medical admission, shock as a cause of admission, sepsis diagnosis, ICU length of stay, serum albumin values at ICU discharge and maximum SOFA score. APACHE II score, age and maximum SOFA score did not fulfill the Cox PH criteria and therefore required the use of splines. The results of the final model based on the AIC are shown in Table 2 and Figs. 2 and 3. The model had good discrimination, maintaining the area under the ROC curve at nearly 0.80 over time (Additional file 1: Figure S4).

**Table 2:** Multivariate analyses using a flexible Cox model for variables affecting 1-year survival after ICU discharge

	All patients (*n* = 690)		Patients without organ failure at ICU discharge (*n* = 561)	
Variable	HR (95 % CI)	*P* value	HR (95 % CI)	*P* value
Age	Fig. 2A	<0.001	Additional file 1: Figure S6a	<0.001
COPD	1.79 (1.15–2.79)	0.010	Not included^a^	–
Cancer	1.85 (1.25–2.75)	0.002	1.89 (1.18–3.01)	0.008
SOFA score	Fig. 3A ^b^	<0.001	Additional file 1: Figure S6b^c^	<0.001
Albumin at discharge^d^	0.96 (0.94–0.99)	0.005	0.96 (0.93–0.99)	0.010

*CI* confidence interval, *COPD* chronic obstructive pulmonary disease, *HR* hazard ratio, *SOFA* Sequential Organ Failure Assessment

^a^COPD was not retained in the final model in the sensitivity analysis, with 561 patients discharged without organ failure

^b^Refers to maximum SOFA score observed during ICU stay, entered as a continuous variable and modeled as a non-linear term

^c^Refers to SOFA score at ICU discharge, entered as a continuous variable and modeled as a non-linear term

^d^Change in hazard ratio estimated for one unit change in albumin level measured in g/L. Albumin was modeled as a linear term in the Cox model

The non-linear and time-varying effects of age and maximum SOFA score are shown in Figs. 2 and 3. The impact of organ failures, assessed by maximum SOFA score, tended to decrease over time.

At 24 h before ICU discharge, only 129 patients (19 %) had any organ failure (at least one SOFA score ≥3 in each domain). We therefore conducted a subset analysis using the remaining 561 patients (81 %) discharged without organ failure over the previous 24 h. Three hundred nine (55 %) of these patients had at least one organ failure during their ICU stay. At ICU discharge, 78 % (435 of 561) had at least one residual organ dysfunction [median, 1 (IQR, 1–2) systems with residual organ dysfunction]. After modeling using the same approach as described above, the number of residual organ dysfunctions remained independently associated with reduced survival at 1 year following ICU discharge (Table 2). The impact of residual organ dysfunction was more pronounced when three or more residual organ dysfunctions were present at ICU discharge (Additional file 1: Figure S5).

### One-year health care use

Of 690 patients discharged from the ICU, 565 patients (82 %) were discharged alive from the hospital after a median of 13 [6–28] days. Health care use in the year following discharge is shown in Table 3 and Fig. 1. After hospital discharge, 52 % of the patients visited any emergency department of the hospital at least once, and 37 % of patients were readmitted to the hospital after the index hospitalization, with 40 % occurring after an emergency department visit. Furthermore, 90 % of the patients attended outpatient clinic visits in several specialties during the year after ICU discharge. At 30 days post-discharge, contact with the health care system occurred for 32 % because of readmission, 41 % because of emergency department visits and 75 % because of outpatient clinic visits (Additional file 1: Figure S7).

**Table 3:** Health care resource use in the year following ICU discharge

Post-ICU facilities	Patients (*n* = 690)
Ward	530 (77 %)
Step-down unit	160 (23 %)
Unplanned ICU admission	121 (18 %)
Hospital LOS after ICU discharge, days	
Mean ± SD	23 ± 28
Median [IQR] (range)	13 [6–28] (1–186)
In-hospital mortality	125 (18 %)
Hospital discharge disposition	Patients (*n* = 565)
Long-term acute care facility	20 (4 %)
Rehabilitation facility	27 (5 %)
Home	518 (91 %)
1-yr health resource use	Patients (*n* = 534)^a^
Hospital readmissions	199 (37 %)
Admission from emergency department^b^	79 (40 %)
Days to the first readmission	
Mean ± SD	100 ± 96
Median [IQR] (range)	69 [17–154] (1–360)
Hospital LOS on the first readmission,	
Mean ± SD	12 ± 16
Median [IQR] (range)	6 [3–16] (1–106)
Number of readmissions, median [IQR] (range)	1 [1–2] (1–8)
Emergency department visit	276 (52 %)
Days to the first visit	
Mean ± SD	84 ± 92
Median	49 [14–121] (1–361)
Number of emergency visits, median [IQR] (range)	2 [1–3] (1–27)
Outpatient consultation	478 (90 %)
Days to the first consult	
Mean ± SD	29 ± 45
Median [IQR] (range)	15 [7–321] (1–356)
Number of outpatient visits, median [IQR] (range)	7 [3–13] (1–48)
Day hospital visit	38 (7 %)
Laboratory/radiologic examinations	270 (51 %)
Psychological/psychiatric service visit	58 (11 %)
Rehabilitation outpatient service^c^	77 (14 %)
Long-term acute care facility/rehabilitation^d^	16 (3 %)
1-yr mortality	46 (9 %)

*ICU* Intensive Care Unit, *IQR* interquartile range, *LOS* length of stay, *SD* standard deviation

^a^There were 34 patients who were not treated in our facilities after discharge

^b^Refers to the number of the first hospital readmission occurring after an emergency department visit

^c^Includes physiotherapy, nutrition and speech therapist

^d^Patients who were transferred to a long-term acute care or rehabilitation facilities after hospital readmission

**Fig. 4:**
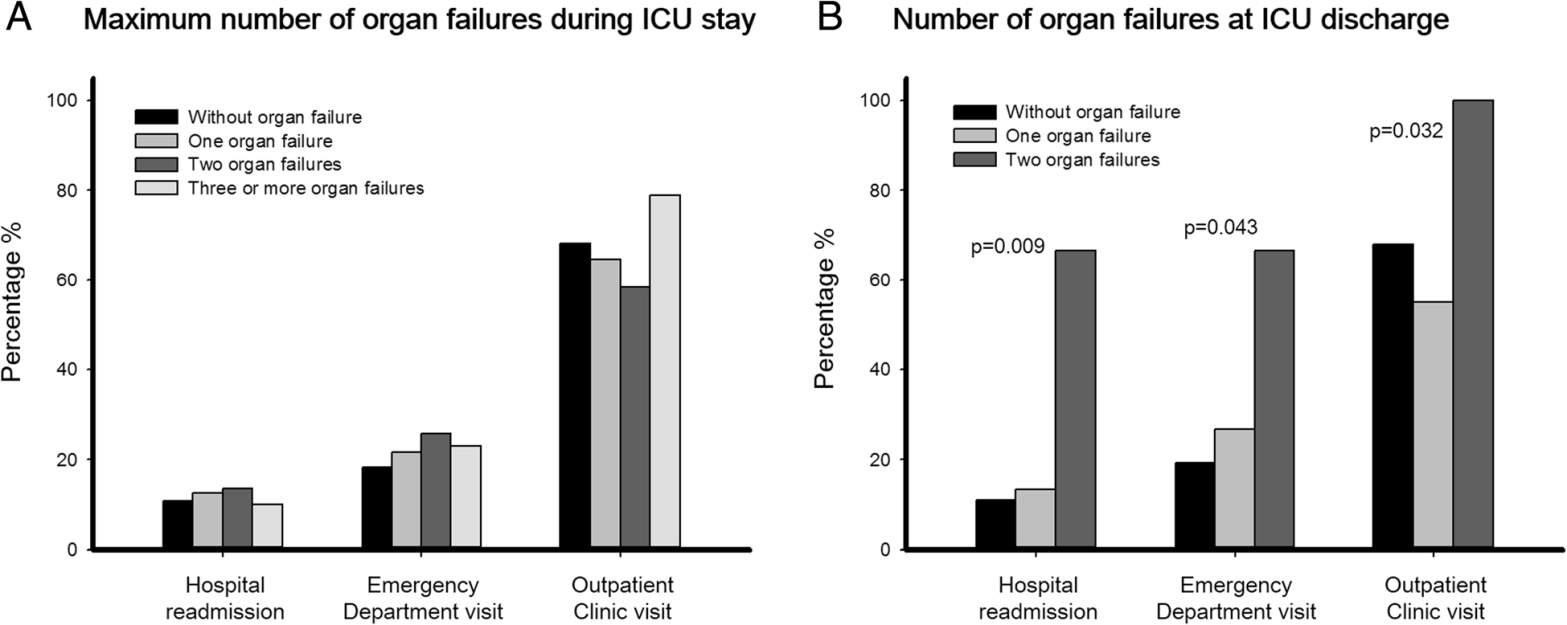
Association between the number of organ failures and health care use in the first 30 days after hospital discharge. (**a**) Crude associations among the maximum numbers of organ failures [evaluated on the basis of the maximum Sequential Organ Failure Assessment (SOFA) score] during the Intensive Care Unit (ICU) stay. (**b**) Crude associations among the numbers of organ failures at ICU discharge (evaluated on the basis of the SOFA score at ICU discharge)

Two hundred seventy patients (51 %) visited the service for complementary examinations, and 14 % attended outpatient rehabilitation services, consisting of physiotherapy, nutrition and speech therapy. Fifty-eight patients (11 %) were followed by the psychological/psychiatric service, and 7 % used a day hospital facility. Use of health resources within the first 30 days after hospital discharge was associated with the number of organ failures at ICU discharge (Fig. 4), but not with the number of maximum organ failures during the ICU stay. For 1-year use of health resources, the number of organ failures had minimal impact (Additional file 1: Figure S8).

## Discussion

In our analysis of 690 patients discharged after at least a 72-h stay in the ICU, we found that 27 % of them died within the first year after discharge and that acquired organ failure had an important and non-linear effect on mortality risk. Additionally, a complementary analysis showed an important burden of these patients on a tertiary public health care complex in Brazil.

Mortality after ICU discharge is a well-known phenomenon in the medical literature, and it probably results from the interplay between patient features (e.g., age, baseline performance status) and the severity of the illness [44]. In this analysis, after choosing ICU discharge as the starting point, we demonstrated that long-term prognosis was associated with the degree of organ failure during ICU stays. Moreover, we emphasized that these effects were non-linear and tended to decrease during the year following ICU discharge. We also report that the number of patients with residual organ dysfunction at the time of ICU discharge was significant and that these dysfunctions had an independent long-term prognostic impact. We believe that this study is the first to focus on the long-term outcomes of a subset of critically ill patients with residual organ dysfunction.

The impact of organ failure on post-ICU mortality has been evaluated previously [1, 8, 25, 26, 28, 44, 45], although little is known about residual organ dysfunction. Lone et al. [25] evaluated the impact of organ failure on 5-year mortality and found that both individual organ failures and the total burden of organ failures (measured by the sum of the worst SOFA score on each domain during the ICU stay) were associated with poorer prognosis, using ICU admission as the starting point for follow-up. The authors performed a landmark analysis, choosing several cut points during the 5-year follow-up period and excluding patients who died before each point [46]. Our study presents a further step of allowing for non-linear and time-varying effects of organ failure and choosing ICU discharge as the starting point. Therefore, we could evaluate an intermediate period during which the patients were at increased risk for mortality (e.g., lower risk than during the ICU stay but higher than 90 days later), which is not considered in the landmark method. Using this approach, we found that the impact of organ failure vanishes over time [14, 28], which could help in the decision-making process, providing information for a period that is amenable to interventions while the patient is in the hospital.

An important finding of our study was the association between residual organ dysfunction at ICU discharge and 1-year mortality. The association between residual organ dysfunction and worse 1-year survival was clear if at least three residual organ dysfunctions were present (Additional file 1: Figure S5). This finding emphasizes the additive effect of residual organ dysfunction on prognosis. Indeed, a SOFA value of 3, for example, could reflect the presence of one organ failure or three organ systems with a residual degree of dysfunction. Mild degrees might reflect a high burden of residual organ dysfunction owing to increased severity of illness (thereby impacting long-term prognosis), but it might also be consequence of early discharge. The pressure for available beds could play a role in the discharge policy, and interventions in this period have been suggested to decrease the risk of death following ICU discharge [47]. Quantifying residual organ dysfunction at discharge might offer a window of opportunity to identify patients at increased risk for long-term mortality.

Health care use by ICU survivors has been described recently for mechanically ventilated [16, 48], septic [49] and elderly [18] critically ill patients in the United States, for the general population in Canada [19] and for patients in 22 ICUs in the United Kingdom [21]. To the best of our knowledge, the present study is the first of the burden of ICU survivors in a developing country with a national universal public health coverage system. Our results reinforce the special attention required for these patients during the post-hospitalization period. Almost 40 % of patients were rehospitalized, and 52 % visited the emergency department at least once within the first year after discharge, similar to the burden reported in Canada [19]. The number of organ failures was indicative only of early health care use. For long-term health care use, our analysis was underpowered because it was not possible to account for higher use of resources by patients who died earlier [50].

One of the strengths of our study is that it allowed for non-linear and time-varying effects [29]. If one aims to develop interventions that impact long-term prognosis after critical illness, the discharge period might represent the most optimal time to initiate an intervention to modify long-term outcomes. To develop the correct approach after discharge, it is imperative to know how risk factors for long-term mortality behave over time. This behavior can be assessed only if the statistical analysis considers time, which is impossible in common logistic regression and traditional Cox models [14, 39].

Nevertheless, this study had some limitations that must be acknowledged. First, it was a single-center analysis; as such, it was subject to local bias, and it had results that were potentially not generalizable. Second, we did not have data on health care use outside our health care complex by these patients; therefore, we could have underestimated their burden. We speculate that this underestimation was low because these patients had a high rate of follow-up (93 %) and a significant measured burden after discharge (e.g., 90 % of them presented at an outpatient clinic, with a median of 7 [3–13] outpatient visits in the first year). Third, our sampling was by convenience, and it could have been underpowered. Fourth, organ failure was measured using the SOFA score, which does not measure other signs of organ dysfunction that might be associated with poor prognosis [51–54]. As previously noted by Lone and Walsh [25], the SOFA score can also measure comorbidities (e.g., chronic renal failure on dialysis or cirrhosis), which could influence the results to some degree. Nevertheless, this theory does not explain the reduced impact of the SOFA score over time. Fifth, we did not have data regarding comorbidities such as Charlson or Elixhauser comorbidity index scores, which might have reduced our ability to explore comorbidities more comprehensively. Sixth, we had no data regarding the health care burden during the period before ICU admission [20] and no data from a control group [18]. Finally, we could not represent the private health system in Brazil.

## Conclusions

Both the maximum number of organ failures during ICU stays and the presence of residual organ dysfunction at discharge were associated with poor prognosis in ICU survivors. These associations tended to decrease over time. The use of health care resources was high among ICU survivors, and target actions are necessary to deliver appropriate care for the improvement of the long-term burden of critical illness.

## Key messages

- Organ failure, both in number and intensity, not only represents an acute burden for the critically ill but also impacts 1-year prognosis and resource use after ICU discharge.
- The effects of organ failure on long-term outcomes decrease over time.
- ICU discharge is an important time point in the dynamic process of critical illness, and it represents an opportunity to plan actions and to improve long-term outcomes.

## Additional file

Additional file 1:
**Complementary figures showing study flowchart, methodology and results.**

## Abbreviations

AIC: Akaike Information Criterion
APACHE II: Acute Physiology and Chronic Health Evaluation II
CI: Confidence interval
CNS: Central nervous system
COPD: Chronic pulmonary obstructive disease
ED: Emergency Department
HR: Hazard ratio
ICU: Intensive Care Unit
IQR: Interquartile range
LOS: Length of stay
PH: Proportional hazards
ROC: Receiver operating characteristic
SD: Standard deviation
SOFA: Sequential Organ Failure Assessment
VIF: Variance inflation factor
