# Supplementary material for: One-year survival and resource use after critical illness: impact of organ failure and residual organ dysfunction in a cohort study in Brazil
Source: Crit Care. 2015 Jun 25;19(1):269. doi: 10.1186/s13054-015-0986-6 (PMC4512155; doi:10.1186/s13054-015-0986-6)
Supplement: Additional file 1: — Complementary figures showing study flowchart, methodology and results. [file 13054_2015_986_MOESM1_ESM.pdf]

Electronic supplementary material

One-year survival and resource use after critical illness: impact of organ failure and residual organ dysfunction in a cohort study in Brazil

Otavio T Ranzani, MD; Fernando G Zampieri, MD; Bruno A. M. P. Besen, MD;

Luciano Cesar Pontes Azevedo, MD, PhD; Marcelo Park, MD, PhD

eFigure 1: Study flowchart

eTable 1: Comparison among analyzed cases and losses follow-up within 1 year after ICU discharge

eFigure 2: SOFA score during ICU stay among survivors and not-survivors

eFigure 3: One-year survival curves by number of organ failures evaluated through the maximum SOFA score

eFigure 4: Discrimination of the flexible Cox model for one-year survival

eFigure 5: One-year survival curves by number of residual organ dysfunctions

eFigure 6: Non-linear effects for age and SOFA score at ICU discharge

eFigure 7: First contact with the health care system after hospital discharge among patients with at least one event (A) and cumulative occurrence of the first contact among the entire cohort (B)

eFigure 8: Association between number of organ failures and one-year health care use for maximum failure during ICU stay (A) and at ICU discharge (B)

eFigure 1: Study flow-chart

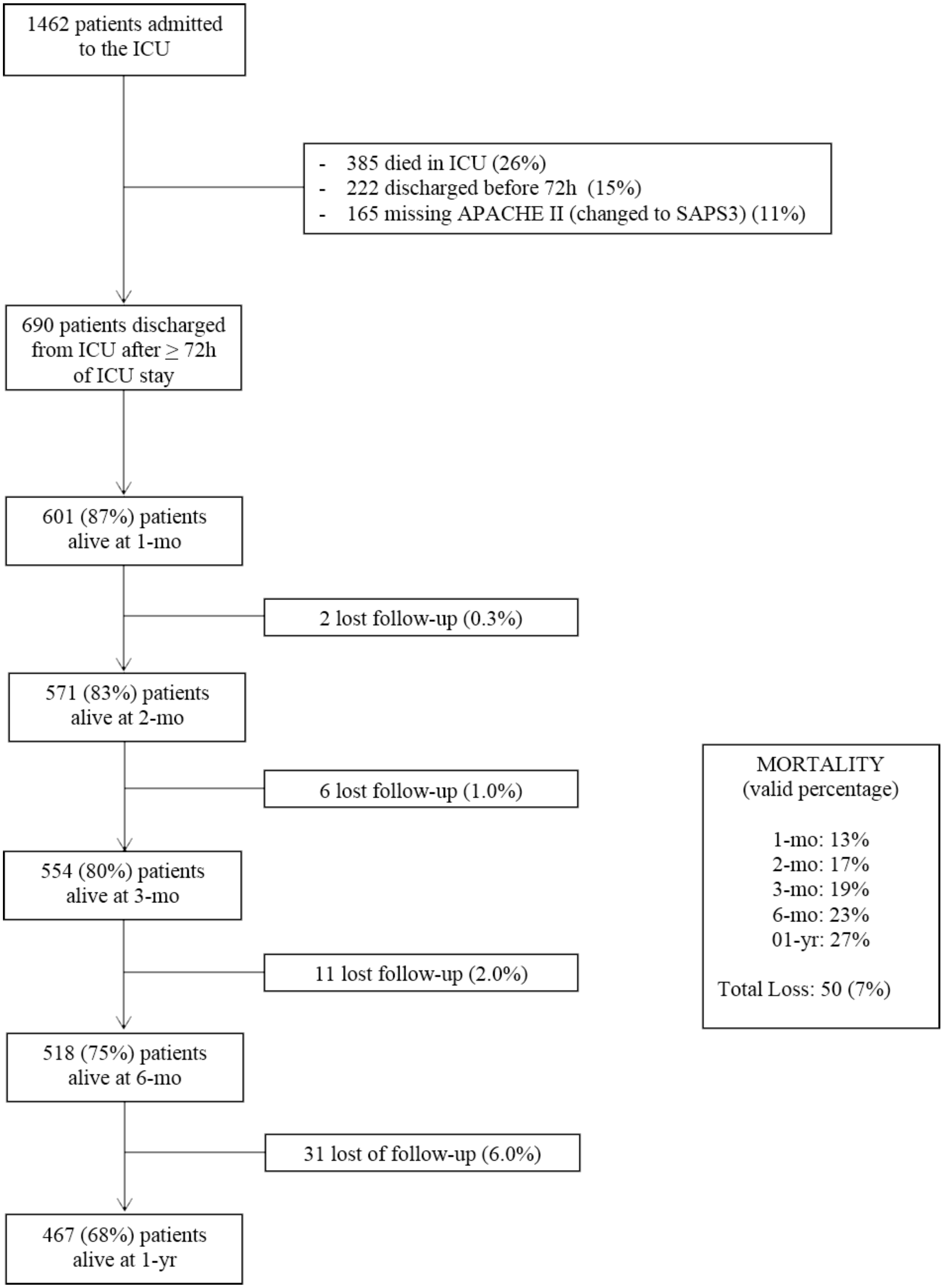

eTable 1: Comparison among analyzed cases and losses follow-up within 1 year after ICU discharge

Variables	All patients (n=690)	Analyzed (n=640)	Lost follow-up (n=50)	P value[#]
**Age, mean years (SD)**	50.3 ± 19	50.3 ± 19	51.4 ± 19	0.705
**Male gender, n (%)**	358 (52%)	330 (52%)	28 (56%)	0.545
**APACHE II**	16 [10-21]	16 [10-21]	14 [8-21]	0.386
**Type of admission, n (%)**				0.434
Medical	550 (80%)	508 (79%)	42 (84%)	
Surgical	140 (20%)	132 (21%)	8 (16%)	
**Comorbidities**				
Number of comorbidities, n	1 [0-2]	1 [0-2]	1 [0-3]	0.658
Hypertension, n (%)	346 (50%)	323 (51%)	23 (46%)	0.543
Diabetes, n(%)	149 (22%)	137 (21%)	12 (24%)	0.668
COPD, n (%)	59 (9%)	54 (8%)	5 (10%)	0.606
Heart failure, n (%)	110 (16%)	102 (16%)	8 (16%)	0.991
Chronic kidney disease, n	113 (16%)	105 (16%)	8 (16%)	0.940
AIDS, n (%)	18 (3%)	15 (2%)	3 (6%)	0.135
Cancer, n (%)	71 (10%)			
**Reason for admission, n (%)**				
Acute respiratory failure	220 (32%)	201 (31%)	19 (38%)	0.335
Shock	125 (18%)	112 (18%)	13 (26%)	0.133
Septic shock	111 (16%)	100 (16%)	11 (22%)	0.237
CNS disorder	78 (11%)	76 (12%)	2 (4%)	0.105
Monitoring	73 (10%)	66 (10%)	7 (14%)	0.414
Post-operative period	48 (7%)	46 (7%)	2 (4%)	0.567
Gastrointestinal diseases	50 (7%)	46 (7%)	4 (8%)	0.777
Electrolyte disturbances	25 (4%)	25 (4%)	-	-
Acute kidney injury	23 (3%)	22 (3%)	1 (2%)	>0.999
Trauma	22 (3%)	21 (3%)	1 (2%)	>0.999
**Support during ICU stay**				
Mechanical ventilation	510 (74%)	468 (73%)	42 (84%)	0.092
Renal replacement therapy	102 (15%)	97 (15%)	5 (10%)	0.410
Vasopressors	281 (41%)	256 (40%)	25 (50%)	0.166
**SOFA score**, median [IQ]				
At admission	4 [2-7]	4 [2-7]	4 [2-8]	0.969
Maximum	6 [3-9]	6 [3-9]	5 [3-10]	0.649
At ICU discharge	2 [1-3]	2 [1-3]	2 [1-3]	0.933
**At least one organ failure***				
At admission	360 (52%)	335 (52%)	25 (50%)	0.749
Maximum	438 (64%)	410 (64%)	28 (56%)	0.254
At ICU discharge	129 (19%)	120 (19%)	9 (18%)	0.896
**ICU length of stay, days**				
Mean ± SD	10 ± 9	10 ± 9	8 ± 5	0.531
Median [IQR]	7 [4-11]	7 [4-12]	6 [4-11]	
**Albumin at discharge (g/L)**	27 [23-31]	27 [23-31]	27 [23-31]	0.853
**Follow-up time, days**				
Mean ± SD	-	-	213 ± 102	
Median [IQR]	-	-	215 [129-310]	

Legend: APACHE II: Acute physiologic and chronic health evaluation; COPD: chronic obstructive pulmonary disease; AIDS: Acquired immunodeficiency syndrome; CNS: Central nervous system. * Organ failure was defined as a value of three or four in a corresponding component of the SOFA score. # P values refers to comparisons between analyzed and losses to follow-up cases. For categorical variables, Fisher's exact test or Chi-squared tests were used; for continuous variables, unpaired t-test or Mann-Whitney test, if normally or non-normally distributed, respectively.

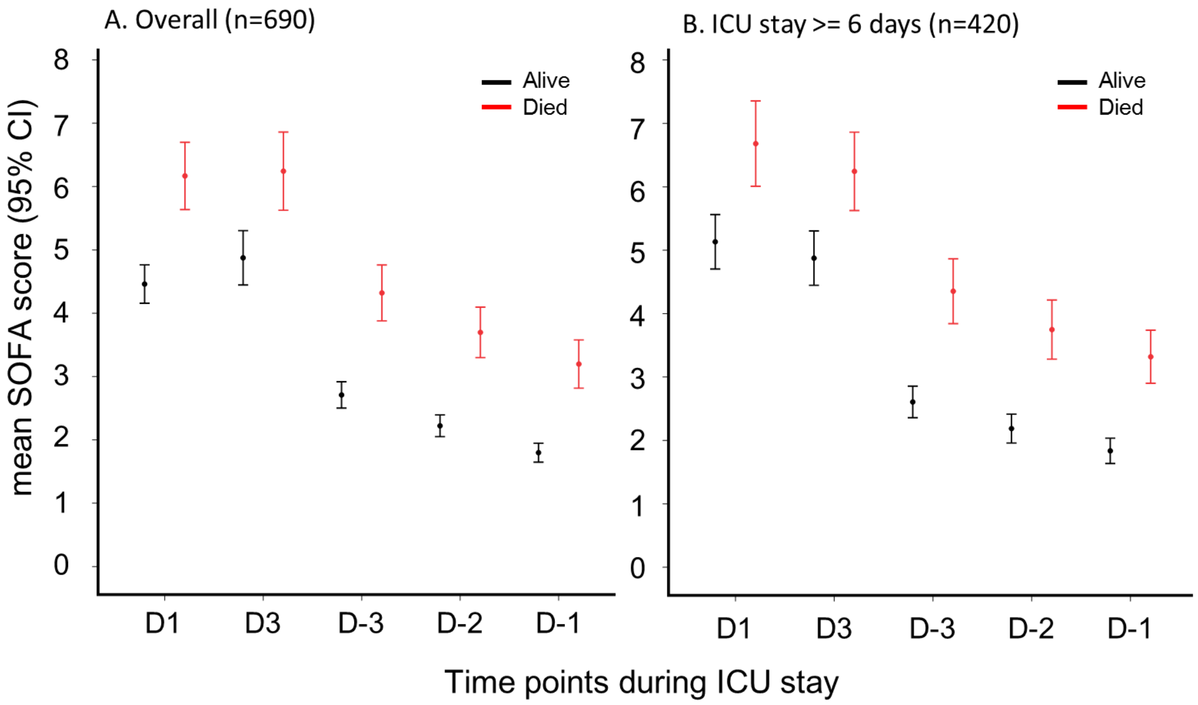

eFigure 2: **SOFA score during ICU stay.** Survivors and non-survivors at one-year after ICU discharge showed similar temporal pattern of SOFA score (p=0.494), with a trend to improvement from admission to ICU discharge (p<0.001). However, patients who died had higher scores during ICU stay (p<0.001) for the entire sample (n=690, panel A). When analyzing only patients who stayed at least 6 days in the ICU (panel B), survivors and non-survivors at one-year after ICU discharge showed similar temporal pattern of SOFA score (p=0.968), with a trend to improvement from admission to ICU discharge (p<0.001). However, patients who died had higher scores during ICU stay (p<0.001). SOFA denotes Sequential Organ Failure Assessment; D1 denotes total SOFA at ICU admission; D3 denotes total SOFA at third day of ICU admission; D-3 denotes total SOFA at 72 hours before ICU discharge time; D-2 denotes total SOFA at 48 hours before ICU discharge time; D-1 denotes the total SOFA within 24 hours before ICU discharge time. CI denotes the Confidence Interval.

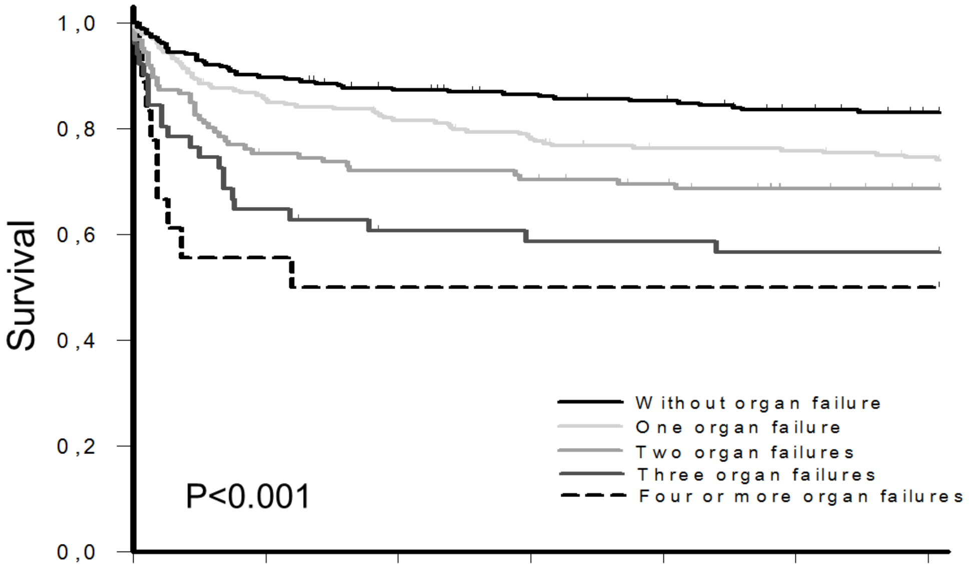

Time (days)	0	60	120	180	240	300	360
No. patients under risk (overall)	690	569	539	518	502	485	467
Without organ failure	262	236	225	217	213	204	200
One organ failure	233	198	188	179	171	170	163
Two organ failures	126	92	88	85	81	75	70
Three organ failures	51	33	29	28	28	27	26
Four or more organ failures	18	10	9	9	9	9	8

eFigure 3: One-year survival curves by number of organ failures evaluated through the maximum SOFA score (one organ failure = SOFA score >=3 in a system) during ICU stay (n=690).

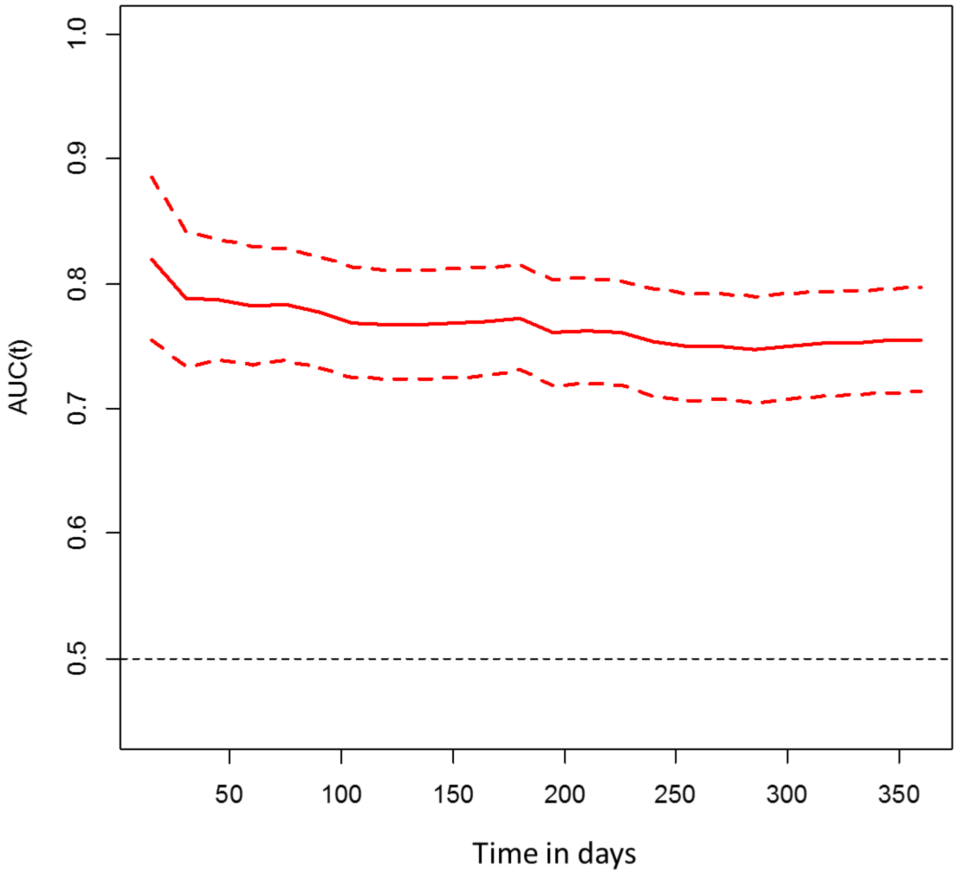

eFigure 4: Discrimination of the flexible Cox model for one-year survival. Time-ROC plot. The solid red line denotes the AUC value for each time point until 365 days. Dashed lines represents 95% CI.

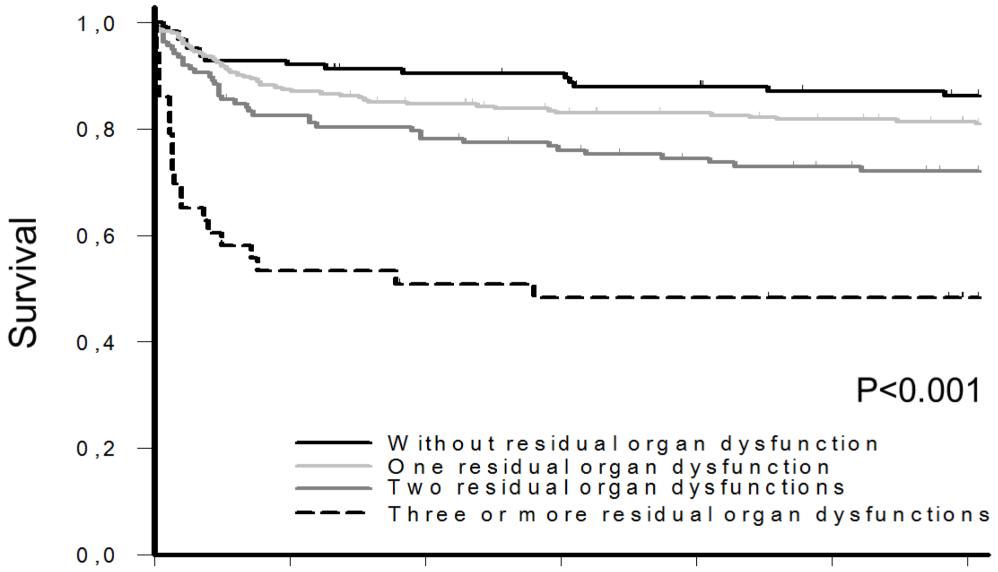

Time (days)	0	60	120	180	240	300	360
No. patients under risk (overall)	561	473	450	435	423	409	394
Without residual organ dysfunction	126	116	111	110	106	102	102
One residual organ dysfunction	254	222	212	204	201	196	189
Two residual organ dysfunctions	138	113	107	102	97	93	88
Three or more residual organ dysfunctions	43	22	20	19	19	18	15

eFigure 5: One-year survival curves by number of residual organ dysfunctions (one residual organ dysfunction = SOFA score 1 or 2 in a system) at ICU discharge among patients without organ failure at discharge (n=561).

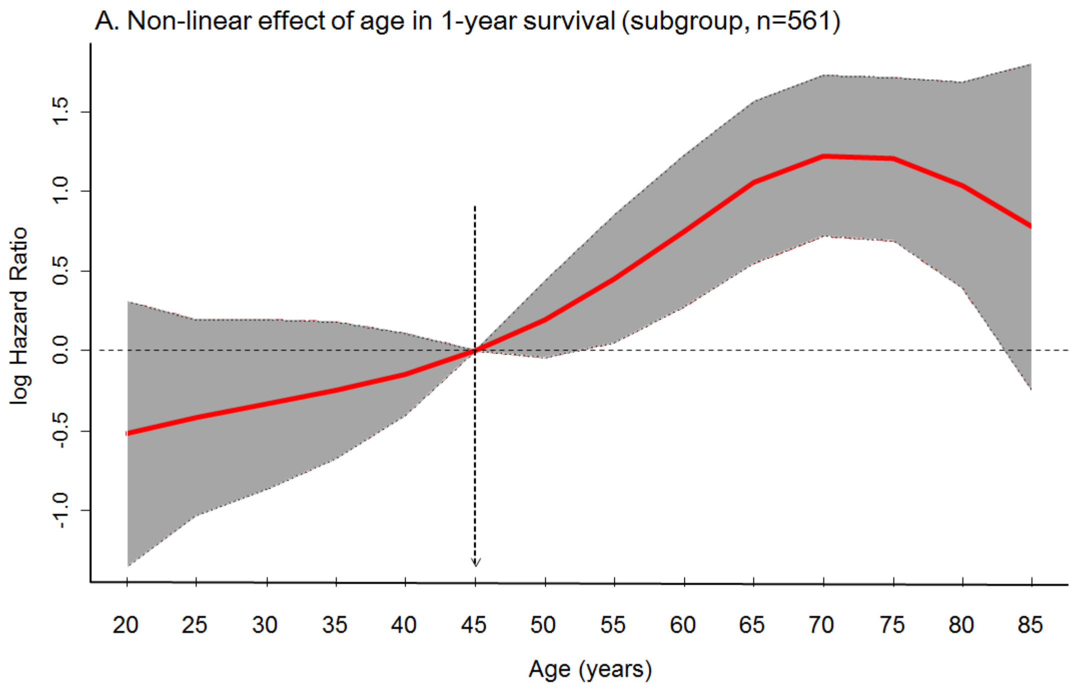

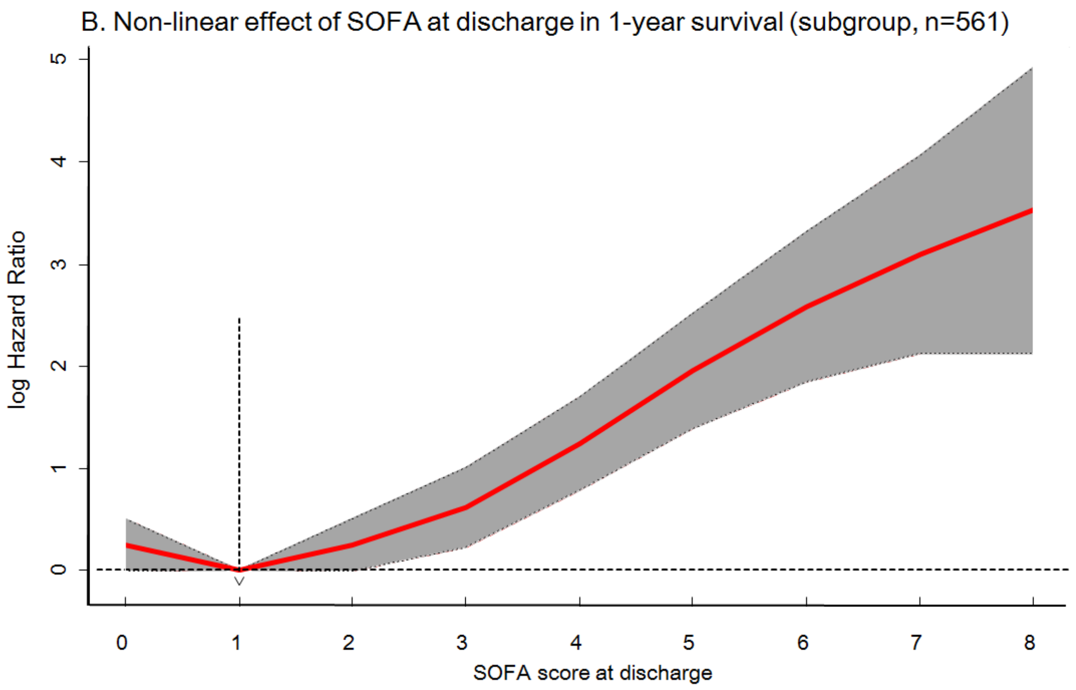

eFigure 6 – Non-linear effects for age (Panel A) and SOFA score at ICU discharge (Panel B) from multivariate flexible Cox model (Table 2) for patients discharged without organ failure (n=561). *Red solid* line is the non-linear effect modeled with spline terms. Grey shaded bands represents 95% CI. Dashed black lines with arrow represent the reference value (mean) for the log Hazard Ratio calculation. The Y axis is in natural log scale, therefore we present some examples of Hazard Ratios (HR) to clinical interpretation: log (-1) = HR of 0.37, log (-0.5) = HR of 0.60, log (0) = HR of 1, log (1) = HR of 2.7 and log (2) = 7.4.

A

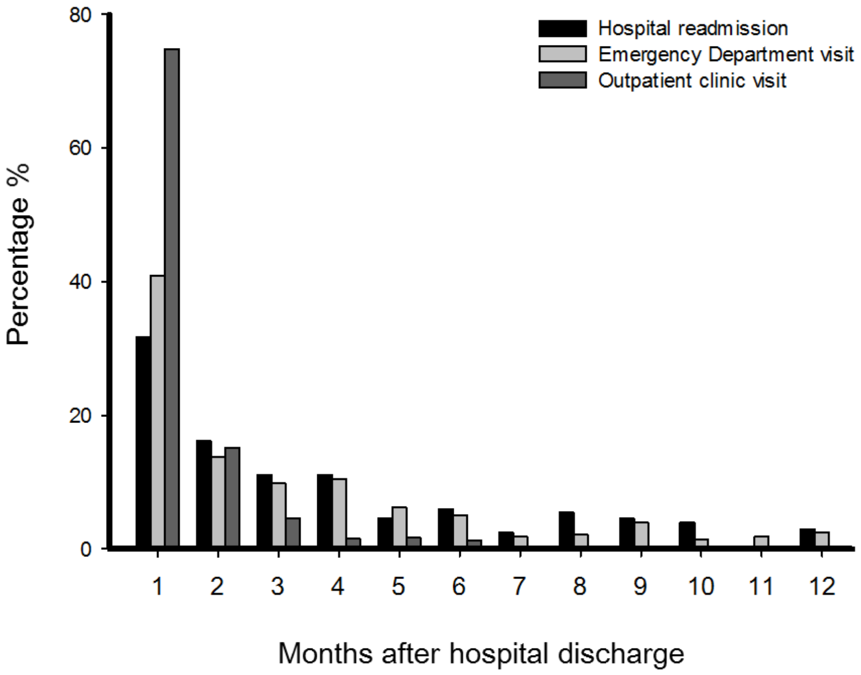

B

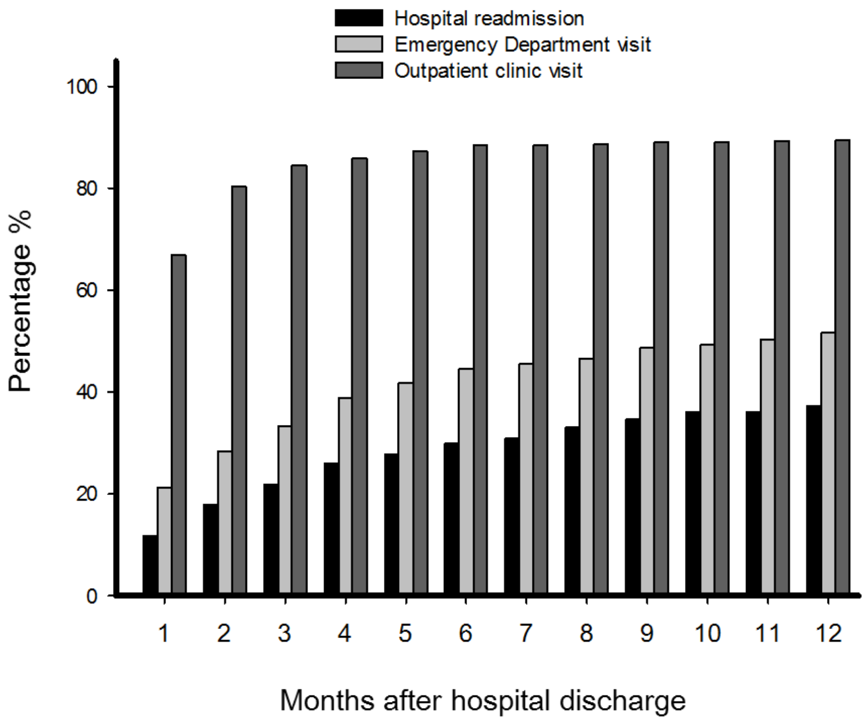

eFigure 7 - First contact with the health care system after hospital discharge among patients with at least one event (A) and cumulative occurrence of the first contact among the entire cohort (B) (n=534)

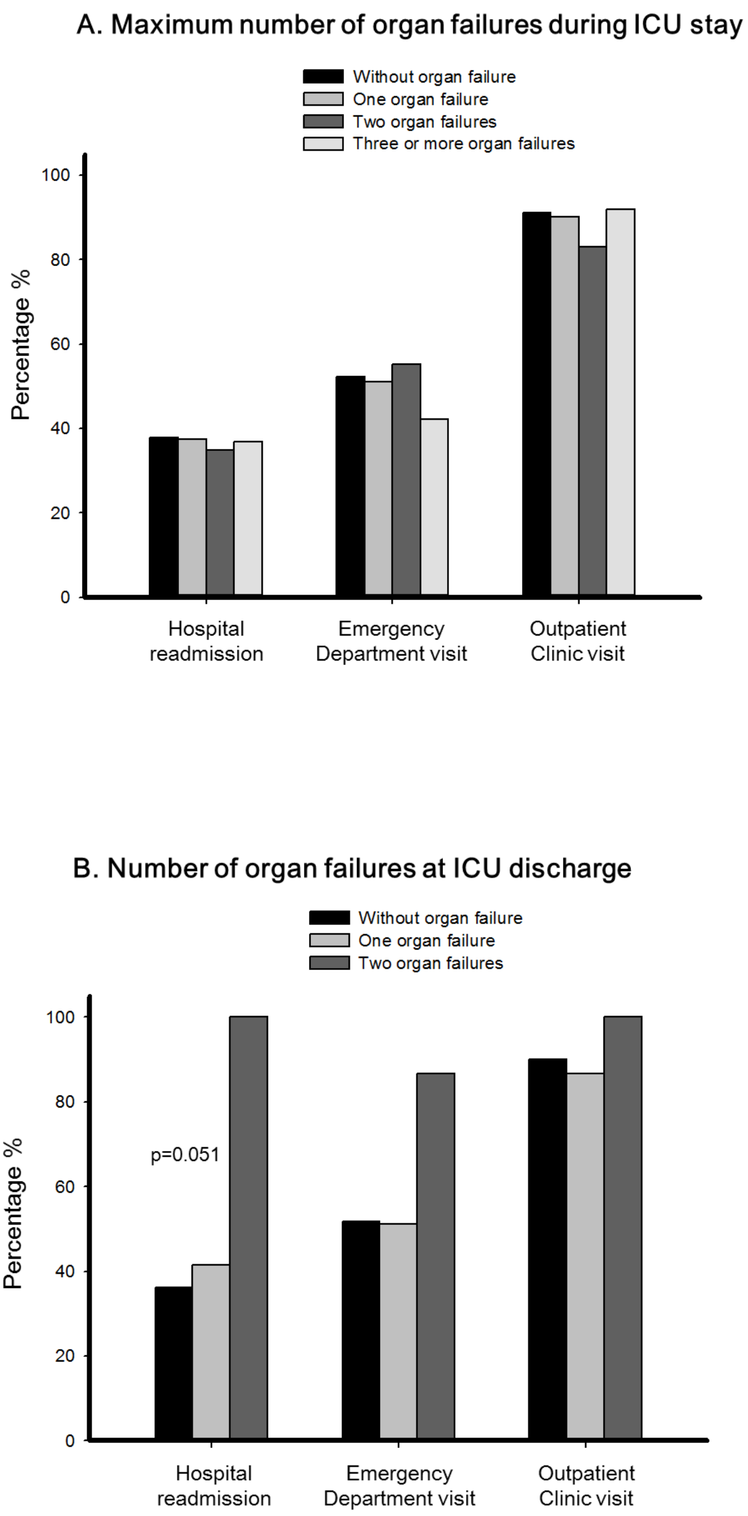

eFigure 8- Association between number of organ failures and one-year health care use for maximum failure during ICU stay (A) and at ICU discharge (B) (n=534)
